# The Effects of Deep Brain Stimulation on Mood and Quality of Life in Parkinson’s Disease: A Systematic Review and Meta-Analysis

**DOI:** 10.7759/cureus.44177

**Published:** 2023-08-26

**Authors:** Nour El Ghazal, Hayato Nakanishi, Alfonso E Martinez-Nunez, Nader K Al Sabbakh, Omotayo A Segun-Omosehin, Natalie E Bourdakos, Maya Nasser, Reem H Matar, Christian Than, Omar A Danoun, Andrew Johnson

**Affiliations:** 1 Neurosurgery, St George's University of London, London, GBR; 2 Neurosurgery, University of Nicosia Medical School, Nicosia, CYP; 3 Neurology, Henry Ford Health System, Detroit, USA; 4 Gastroenterology and Hepatology, Mayo Clinic, Rochester, USA; 5 Biomedical Sciences, The University of Queensland, Brisbane, AUS; 6 Neurological Institute, Northshore Medical Group, Chicago, USA

**Keywords:** non-motor symptoms, parkinson's disease, globus pallidus internus, subthalamic nucleus, deep brain stimulation

## Abstract

Deep brain stimulation (DBS) is extensively used to treat motor and non-motor symptoms in Parkinson’s disease (PD). The aim of this study was to investigate the difference between subthalamic (STN) and globus pallidus internus (GPi) DBS on mood and quality of life with reference to minimal clinically important differences (MCID). A systematic literature search for articles published until November 2022 yielded 14 studies meeting the eligibility criteria, with a total of 1,088 patients undergoing STN (n=571) or GPi (n=517) stimulation. Baseline patient and clinical characteristics were comparable between the two groups. Results showed that GPi stimulation demonstrated a greater reduction in the Beck depression inventory (mean difference (MD)=1.68) than STN stimulation (MD=0.84). Hospital anxiety and depression scale showed a 2.69- and 3.48-point decrease by the GPi group in the depression and anxiety categories, respectively. The summary index (SI) of the PD questionnaire depicted a greater improvement in the GPi group from baseline (mean=41.01, 95% CI 34.89, 47.13) to follow-up (mean=30.85, 95% CI 22.08, 39.63) when compared to the STN group (baseline mean=42.43, 95% CI 34.50, 50.37; follow-up mean=34.21, 95% CI 25.43, 42.99). The emotions category also demonstrated a similar trend. However, STN stimulation showed greater reductions in motor symptoms and medication than GPi stimulation. This meta-analysis demonstrated that GPi stimulation seems to offer an advantage over STN stimulation in improving mood and quality of life in PD, but those effects must be further validated by larger studies.

## Introduction and background

Parkinson’s disease (PD), a rapidly growing neurological disorder and the leading cause of disability [1], is characterized by motor symptoms, such as tremors and rigidity, and non-motor symptoms, such as mood disturbances, cognitive dysfunction, and sleep-wake cycle dysregulations [2]. Non-motor manifestations have been shown to contribute to patients’ chronic burden and disability as they can sometimes precede the occurrence of motor symptoms [2] and be exacerbated by medication used to treat motor symptoms [3]. As a result, new promising therapies are being developed that focus on altering the course of the disease and alleviating motor and non-motor symptoms.

Deep brain stimulation (DBS) is widely used to treat the motor symptoms of PD, and it involves placing electrodes on selected deep nuclei to interfere with pathological oscillations, leading to an informational disruption [4]. The subthalamic nucleus (STN) and the globus pallidus internus (GPi) are the most commonly chosen sites to treat the bradykinetic features of the disease, and they are both FDA-approved targets [5]. This surgical treatment has demonstrated significant improvement in motor symptoms of patients with PD, but research investigating its effects on non-motor symptoms is still gaining traction [6]. In addition, while several studies and randomized controlled trials (RCTs) have highlighted the efficacy of both STN and GPi DBS, most of the current treatments have predominantly chosen the STN as the stimulation target [7] since it provides an advantage in reducing dopaminergic medication doses, which positively affects quality of life [8]. Several studies have demonstrated worse cognitive outcomes, depression, and anxiety with STN-DBS compared to GPi-DBS. Other trials have also highlighted that stimulation of either site is effective in alleviating non-motor symptoms with no significant difference between the two targets [9]. As such, controversy arises when it comes to the selection of the optimal target, and questions remain regarding the effects of DBS on non-motor symptoms, which form an integral part of determining the quality of life of patients following treatment.

For ease of assessment, non-motor manifestations have been categorized into nine domains as reported in the non-motor symptom assessment scale for PD: cardiovascular, sleep/fatigue, mood/cognition, perceptual problems/hallucinations, attention/memory, gastrointestinal tract, urinary, sexual function, and miscellaneous [10]. Previous meta-analyses have studied the effects of STN stimulation on quality of life in general or on one of the specific domains. For instance, Cartmill et al. examined mood changes, which encompass subcategories, such as depression, apathy, and energy. They reported that, after bilateral STN stimulation in patients with PD, there was a significant reduction in depressive symptoms [11]. Another meta-analysis demonstrated that STN DBS is an effective method in the management of sleep quality and restless leg symptoms [12].

However, RCTs comparing the effect of STN and GPi DBS stimulation on non-motor symptoms remain sparse [13], and this also remains true for investigations into depression and quality of life together. To the best of our knowledge, no previous meta-analysis has assessed the effects of STN and GPi stimulation on non-motor outcomes using the minimal clinically important difference (MCID), which is defined as “the smallest change or difference in an outcome measurement that is perceived as beneficial and would lead to a change in the patient’s medical management” [14]. Therefore, the aim of this meta-analysis is to investigate the difference between DBS of the STN and the GPi on mood and quality of life in PD and to identify the presence of any MCID.

## Review

Methods

Data Sources and Search Strategies

A comprehensive search of several databases from inception to November 6, 2022, was conducted in compliance with the Preferred Reporting Items for Systematic Reviews and Meta-analyses (PRISMA) guidelines [15]. The databases included Ovid MEDLINE(R) and Epub Ahead of Print, In-Process & Other Non-Indexed Citations and Daily, Ovid Embase, Ovid Cochrane Central Register of Controlled Trials, Ovid Cochrane Database of Systematic Reviews, and Scopus. The search strategy was designed and conducted by an experienced librarian with input from the study’s principal investigator. Controlled vocabulary supplemented with keywords was used to search for studies describing DBS, Parkinson’s disease, and non-motor symptoms. The actual strategy listing all search terms used and how they are combined is available in the Appendix (Table 4). The review was registered prospectively with PROSPERO (CRD42021267096).

Eligibility Criteria and Quality Assessment

Eligible studies were randomized controlled trials (RCTs) or prospective cohort studies that met the following inclusion criteria: 1) comparative studies of adult participants older than or equal to 18 years with DBS (either STN or GPi) for PD; 2) assessment of non-motor symptoms using questionnaires for mood, anxiety and/or quality of life; and 3) assessment of outcomes for a follow-up period of at least six months. Case reports, case series, conference abstracts and/or abstracts, and articles that were not reported in English were excluded from the study. The quality of each study was independently evaluated by two authors (NE and OAS) using the Newcastle-Ottawa scale [16]. Any discrepancies were discussed by the two independent assessors, with disagreements addressed via an adjudicator (CAT). (Quality assessment results of cohort studies and randomized controlled trials are shown in Tables 5-6 of the appendix, respectively.)

Statistical Analysis

The pooled means and proportions of our two-arm analysis of the study characteristics were analyzed using an inverse variance method for continuous data and the Mantel-Haenszel method for dichotomous data. The pooled means and estimates of our one-arm analysis of outcomes were analyzed using a random-effect generic inverse variance method of DerSimonian and Laird, which assigns the weight of each study based on its variance [17]. The heterogeneity of effect size estimates across the studies was quantified using the Q statistic and the I^2^ index (p<0.10 was considered significant) [18]. A value of I^2^ of 0-25% indicates minimal heterogeneity, 26-50% moderate heterogeneity, and 51-100% substantial heterogeneity. The random-effects model was used when I^2^>50%, and the fixed-effects model was used when I^2^<50% [18]. Publication bias was assessed using a funnel plot [19]. Data analysis was performed using RevMan software version 5.4 (Review Manager (RevMan); Cochrane Collaboration, 2020, Copenhagen, Denmark; and Open Meta analyst software (CEBM, Brown University, Providence, Rhode Island, USA). If mean and standard deviation (SD) were unavailable, the median was converted to mean using the formulas from the Cochrane Handbook for Systematic Reviews of Interventions [20]. In cases of patient overlap where one trial was adopted in several studies, the original or parent study was used for the meta-analysis, while patient information and outcomes across the secondary studies were combined. 

Outcome Assessment and MCID Interpretation

Outcomes measured in this meta-analysis were assessed using the following scales and questionnaires: the Unified Parkinson’s Disease Rating Scale parts I and III (UPDRS-I and UPDRS-III, respectively), where the former evaluates non-motor aspects of experiences of daily living (such as mood, mentation, and behavior) and the latter evaluates motor symptoms [21]; the Parkinson’s Disease Questionnaire (PDQ-39) consisting of eight domains, from which two - summary index (SI) and emotions - were deemed to be relevant for this study (the Beck Depression Inventory (BDI) and the Hospital Anxiety and Depression Scale (HADS) consisting of anxiety- and depression-specific subcategories) [22]; and finally the levodopa equivalent daily dose (LEDD) to measure medication intake before and after the intervention. MCIDs obtained for this meta-analysis were 3.3 points for BDI [23]; 1.72 and 1.84 points for HADS depression and anxiety, respectively [23]; -4.7 for improvement and 4.2 points for worsening on PDQ-39 [23]; and 3.25 points for UPDRS-III [23].

Results

Study Selection and Patient Characteristics

**Figure 1:**
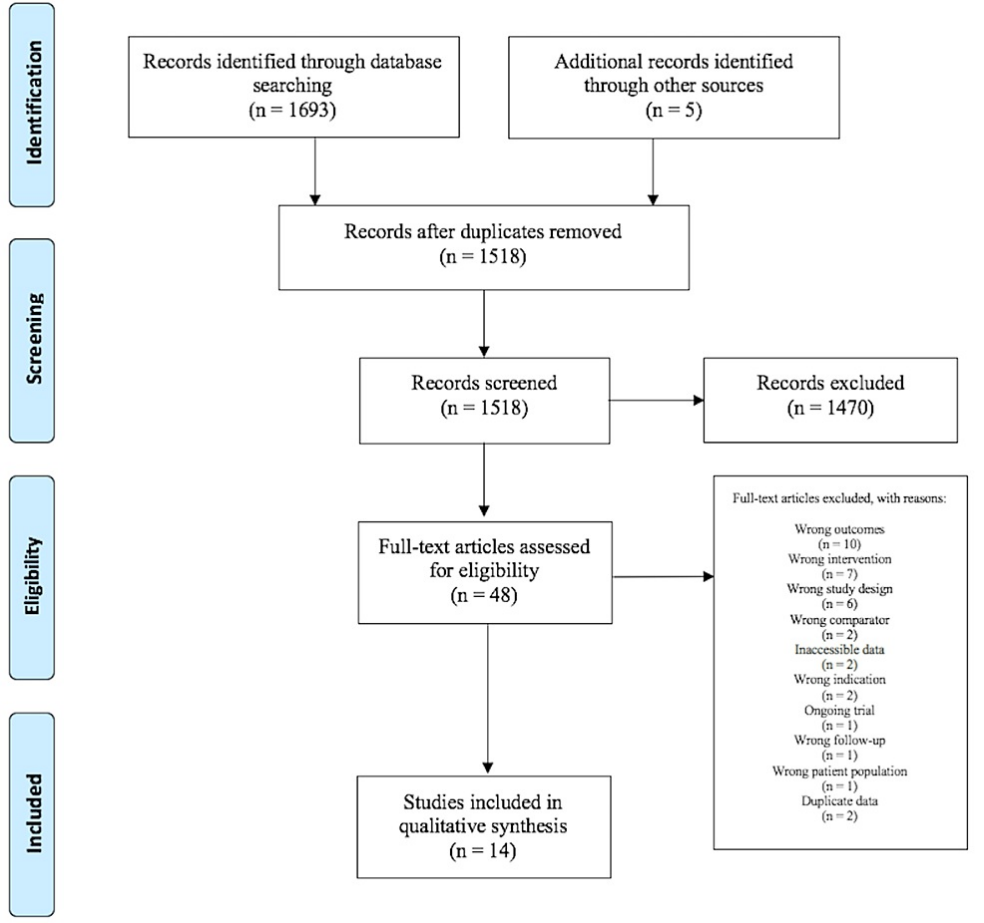
PRISMA flow diagram PRISMA: Preferred Reporting Items for Systematic Reviews and Meta-Analyses

The initial search yielded 1,698 potentially relevant articles, from which full texts of 48 studies were evaluated to finally result in 14 unique studies, meeting the eligibility criteria. Details of the study selection process are depicted in the PRISMA flowchart, as shown in Figure 1.

**Figure 2:**
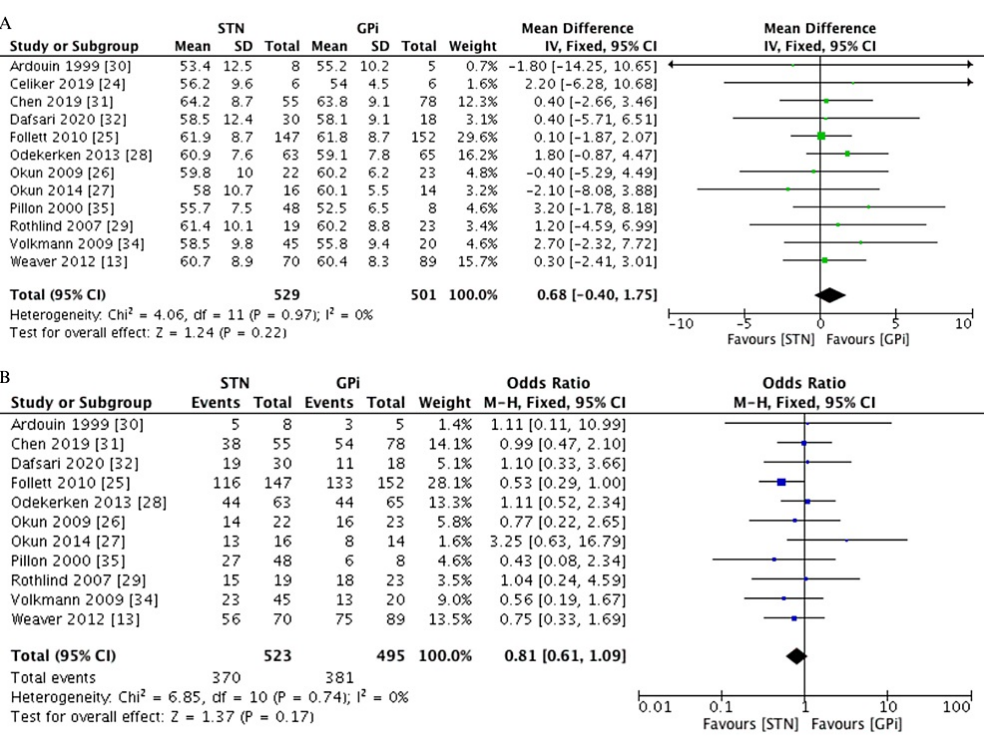
Pooled data of baseline characteristics between the STN and GPi groups. A. Age (years), B. Number of males (n) GPi: globus pallidus internus, STN: subthalamic nucleus

The included studies consisted of seven RCTs [13,24-29], six prospective cohort studies [30-35], and one combining RCT and cohort study designs [36]. A total of 1,088 patients were included and divided between 571 in the STN group (64.8% males) and 517 in the GPi group (73.7% males). The mean age of each group was comparable (MD=0.68 years, 95% CI -0.40, 1.75, I^2^=0%), and the average age of participants across each study ranged from 55.1 to 66.1 years. The baseline characteristics of the included studies are depicted in Table 1 and Figure 2.

**Table 1:** Baseline characteristics of the included studies BDI: Beck depression inventory, GPi: globus pallidus internus, HADS: hospital anxiety and depression scale, LEDD: levodopa equivalent daily dose, n: sample size, NMSQ: non-motor symptoms questionnaire, NMSS: non-motor symptoms scale, NR: not reported, NRCT: non-randomized controlled trial, PDQ: Parkinson’s Disease questionnaire, RCT: randomized controlled trial, STN: subthalamic nucleus, UPDRS: Unified Parkinson’s Disease rating scale

Study authors	Year	Country	Study type	Number of participants (n)	Number of males (n)	Stimulation location (STN:GPi)	Outcomes assessed	Follow-up periods (months)
Weaver et al. [13]	2012	USA	RCT	159	131	70:89	LEDD, PDQ-39, UPDRS-I, UPDRS-III	6, 24, 36
Celiker et al. [24]	2019	Turkey	RCT	12	2	6:6	HADS, LEDD, UPDRS-III,	6, 12, 24
Follett et al. [25]	2010	USA	RCT	299	249	147:152	BDI, PDQ-39, LEDD, UPDRS-I, UPDRS-III	6, 24
Okun et al. [26]	2009	USA	RCT	45	30	22:23	BDI, LEDD, PDQ-39, UPDRS-III	7
Okun et al. [27]	2014	USA	RCT	30	21	16:14	BDI, LEDD, UPDRS-III	12
Odekerken et al. [28]	2013	Netherlands	RCT	128	88	63:65	LEDD, HADS, UPDRS-III	12, 36
Rothlind et al. [29]	2007	USA	RCT	42	33	19:23	BDI, LEDD	6, 15
Ardouin et al. [30]	1999	France	Cohort	13	8	8:5	BDI, LEDD, UPDRS-I, UPDRS-III,	6
Chen et al. [31]	2019	USA	Cohort	133	92	55:78	LEDD, PDQ-39, UPDRS-III	6
Dafsari et al. [32]	2020	Germany	Cohort	48	30	30:18	LEDD, NMSS, PDQ-39, UPDRS-I, UPDRS-III	6, 12, 18, 24
Hwynn et al. [33]	2011	USA	Cohort	10	NR	9:1	NMSS, NMSQ	6
Volkmann et al. [34]	2009	Germany	Cohort	65	36	45:20	UPDRS-III	6, 36
Pillon et al. [35]	2000	France	Cohort	56	33	48:8	BDI, LEDD, UPDRS-III	6, 12
Kirsch-Darrow et al. [36]	2011	USA	RCT + cohort	48	36	33:15	LEDD, UPDRS-III,	6

Risk of Bias

Results of the quality assessment of all included studies are shown in the Appendix (Tables 5-6). All RCTs and prospective cohort studies were judged to be of fair quality. The patients appeared to represent the whole experience of the intervention, the exposure and outcome were adequately ascertained, and the length of follow-up was adequate.

Clinical Characteristics

**Figure 3:**
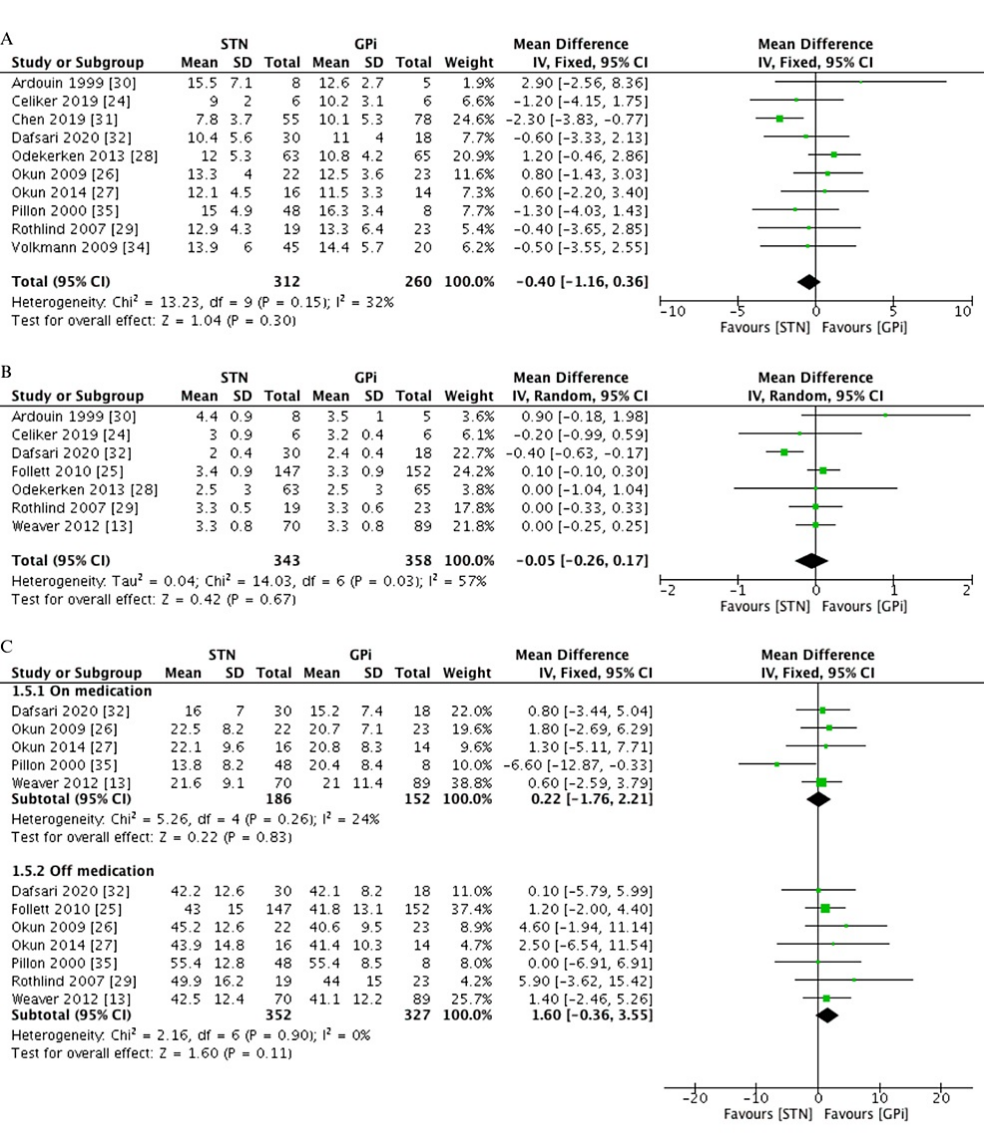
Pooled data of the clinical characteristics between the STN and GPi groups. A. Duration of disease (years), B. Severity of disease (Hoehn-Yahr stage), C. Baseline UPDRS-III on and off medication GPi: globus pallidus internus, STN: subthalamic nucleus, UPDRS: unified Parkinson's disease rating scale

The 14 eligible studies yielded a total of 1,088 patients divided into the STN and GPi groups. The duration of disease experienced by each patient (MD=-0.40 years, 95% CI -1.16, 0.36, I^2^=32%) and the severity of the condition (MD=-0.05, 95% CI -0.26, 0.17, I^2^=57%), which was measured by the Hoehn and Yahr scale, were similar between the two groups. Moreover, motor symptoms at baseline were assessed using the UPDRS-III in the off-medication (MD=1.60, 95% CI -0.36, 3.55, I^2^=0%) and the on-medication state (MD=0.22, 95% CI -1.76, 2.21, I^2^=24%), and no difference was found between the two groups. In addition, assessment of stimulation parameters revealed the following: while pulse width (MD=-13.07 µsec, 95% CI -29.90, 3.76, I^2^=94%) and frequency (MD=-3.97 Hz, 95% CI -8.31, 0.36, I^2^=0%) were similar between both groups, patients undergoing STN stimulation were subjected to a lower voltage than those undergoing GPi stimulation (MD=-0.45 V, 95% CI -0.62, -0.28, I^2^=33%). The follow-up periods ranged from six to 36 months, and when the number of studies included in each follow-up period was inadequate for pooling, outcomes over several follow-up periods were combined to express the furthest timeframe in each study. A summary of the clinical characteristics of the included studies is described in Table 2 and Figure 3.

**Table 2:** Clinical characteristics of the included studies † Odekerken et al. 2013 [28]: stimulation parameters were measured at follow-up only ‡ Okun et al. 2009 [26]: Hoehn and Yahr staging calculated from reported percentages, and no SD was given GPi: globus pallidus internus, Hz: hertz, n: sample size, NR: not reported, SD: standard deviation, STN: subthalamic nucleus, UPDRS: Unified Parkinson’s Disease rating scale, V: volts

Study authors and year	Number of participants (n)	Mean age (years ± SD)	Duration of disease (years ± SD)	Severity of disease (Hoehn-Yahr stage ± SD)		Stimulation parameters						Baseline UPDRS-III (±SD)			
						Voltage (V ± SD)		Frequency (Hz ± SD)		Pulse width (µsec ± SD)		Off medication		On medication	
				STN	GPi	STN	GPi	STN	GPi	STN	GPi	STN	GPi	STN	GPi
Ardouin et al. 1999 [30]	13	54.09 ± 11.25	14.38 ± 5.83	4.4 ± 0.9	3.5 ± 1.0	2.4 ± 0.7	3.1 ± 0.6	137.0 ± 27.6	139.6 ± 20.6	60.5 ± 10.9	78.5 ± 28.8	NR	NR	NR	NR
Celiker et al. 2019 [24]	12	55.08 ± 7.24	9.58 ± 2.57	3.0 ± 0.9	3.2 ± 0.4	NR	NR	NR	NR	NR	NR	NR	NR	NR	NR
Chen et al. 2019 [31]	133	63.94 ± 8.89	9.12 ± 4.84	NR	NR	2.9 ± 0.7	3.1 ± 0.9	159.5 ± 20.2	164.6 ± 25.3	71.1 ± 13.7	79.1 ± 16.8	NR	NR	NR	NR
Dafsari et al. 2020 [32]	48	58.35 ± 11.17	10.70 ± 5.02	2.0 ± 0.4	2.4 ± 0.4	NR	NR	NR	NR	NR	NR	42.2 ± 12.6	42.1 ± 8.2	16.0 ± 7.0	15.2 ± 7.4
Follett et al. 2010 [25]	299	61.50 ± 8.47	NR	3.4 ± 0.9	3.3 ± 0.9	NR	NR	NR	NR	NR	NR	43.0 ± 15.0	41.8 ± 13.1	NR	NR
Hwynn et al. 2011 [33]	10	66.10 ± 7.80	9.90 ± 3.00	NR	NR	NR	NR	NR	NR	NR	NR	NR	NR	NR	NR
Kirsch-Darrow et al. 2011 [36]	48	60.30 ± 9.00	NR	NR	NR	NR	NR	NR	NR	NR	NR	NR	NR	NR	NR
Odekerken et al. 2013 [28] †	128	59.99 ± 8.41	11.39 ± 4.79	2.5 ± 3.0	2.5 ± 3.0	NR	NR	NR	NR	NR	NR	NR	NR	NR	NR
Okun et al. 2009 [26] ‡	45	60.00 ± 8.20	12.90 ± 3.80	3.0 ± NR ‡	2.8 ± NR ‡	2.4 ± 0.6	2.9 ± 0.4	141.1 ± 13.1	151.5 ± 19.3	94.0 ± 19.1	84.7 ± 14.7	45.2 ± 12.6	40.6 ± 9.5	22.5 ± 8.2	20.7 ± 7.1
Okun et al. 2014 [27]	30	59.00 ± 8.60	11.80 ± 3.90	NR	NR	NR	NR	NR	NR	NR	NR	43.9 ± 14.8	41.4 ± 10.3	22.1 ± 9.6	20.8 ± 8.3
Pillon et al. 2000 [35]	56	55.24 ± 7.40	15.19 ± 4.71	NR	NR	2.4 ± 0.7	3.1 ± 0.6	137.0 ± 27.6	139.6 ± 20.6	60.5 ± 10.9	78.5 ± 28.8	55.4 ± 12.8	55.4 ± 8.5	13.8 ± 8.2	20.4 ± 8.4
Rothlind et al. 2007 [29]	42	60.70 ± 9.33	13.12 ± 5.49	3.3 ± 0.5	3.3 ± 0.6	2.6 ± 0.8	3.3 ± 0.8	185.0 ± 11.5	185.0 ± 11.5	60.0 ± 8.7	92.3 ± 8.7	49.9 ± 16.2	44.0 ± 15.0	NR	NR
Volkmann et al. 2009 [34]	65	57.67 ± 9.69	14.05 ± 5.87	NR	NR	NR	NR	NR	NR	NR	NR	NR	NR	NR	NR
Weaver et al. 2012 [13]	159	60.53 ± 8.54	NR	3.3 ± 0.8	3.3 ± 0.8	NR	NR	NR	NR	NR	NR	42.5 ± 12.4	41.1 ± 12.2	21.6 ± 9.1	21.0 ± 11.4

Non-motor Outcomes

**Figure 4:**
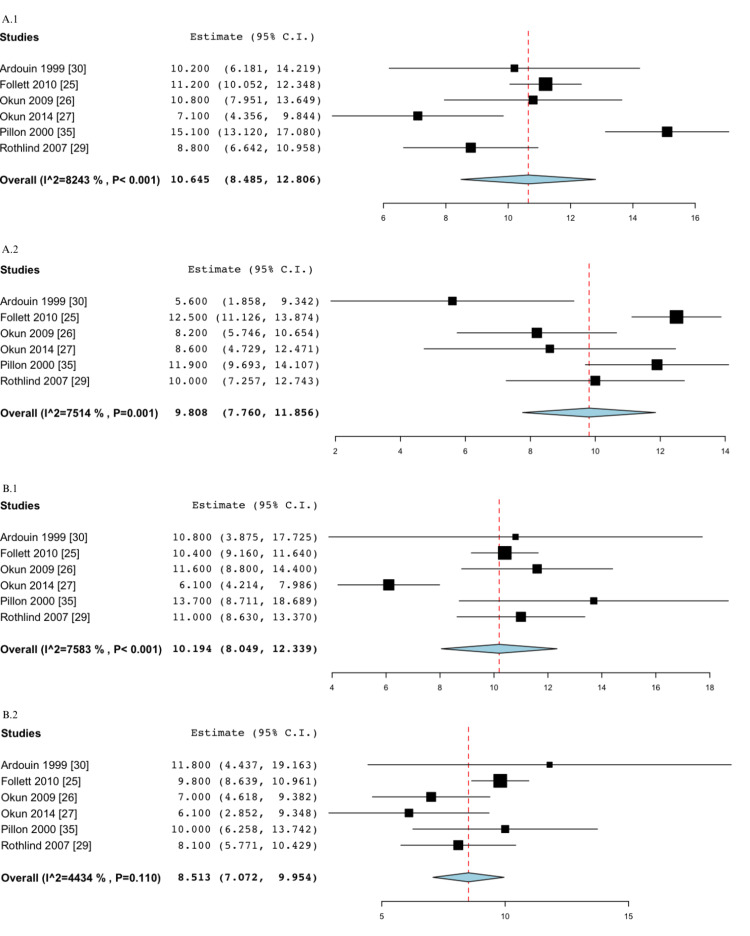
Pooled estimate of BDI outcomes between STN (A) and GPi (B) groups at baseline (A.1, B.1) and six-month follow-up (A.2, B.2) BDI: Beck depression inventory, GPi: globus pallidus internus, STN: subthalamic nucleus

**Figure 5:**
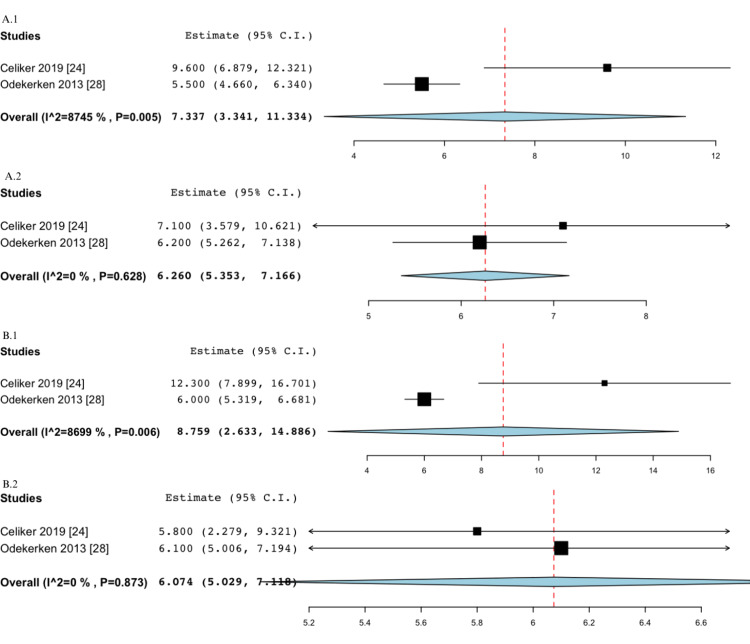
Pooled estimate of HADS depression outcomes between STN (A) and GPi (B) groups at baseline (A.1, B.1) and 12-month follow-up (A.2, B.2) GPi: globus pallidus internus, HADS: hospital anxiety and depression scale, STN: subthalamic nucleus

**Figure 6:**
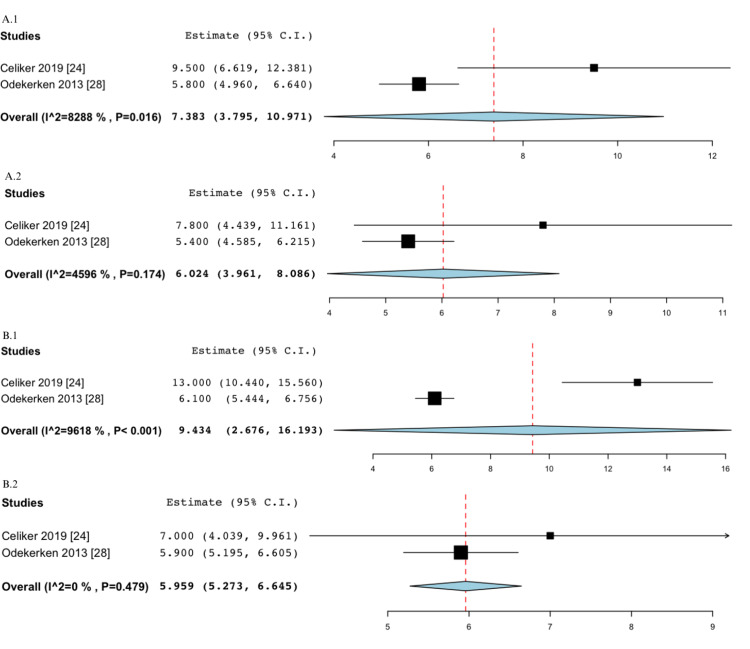
Pooled estimate of HADS anxiety outcomes between STN (A) and GPi (B) groups at baseline (A.1, B.1) and 12-month follow-up (A.2, B.2) GPi: globus pallidus internus, HADS: hospital anxiety and depression scale, STN: subthalamic nucleus

**Figure 7:**
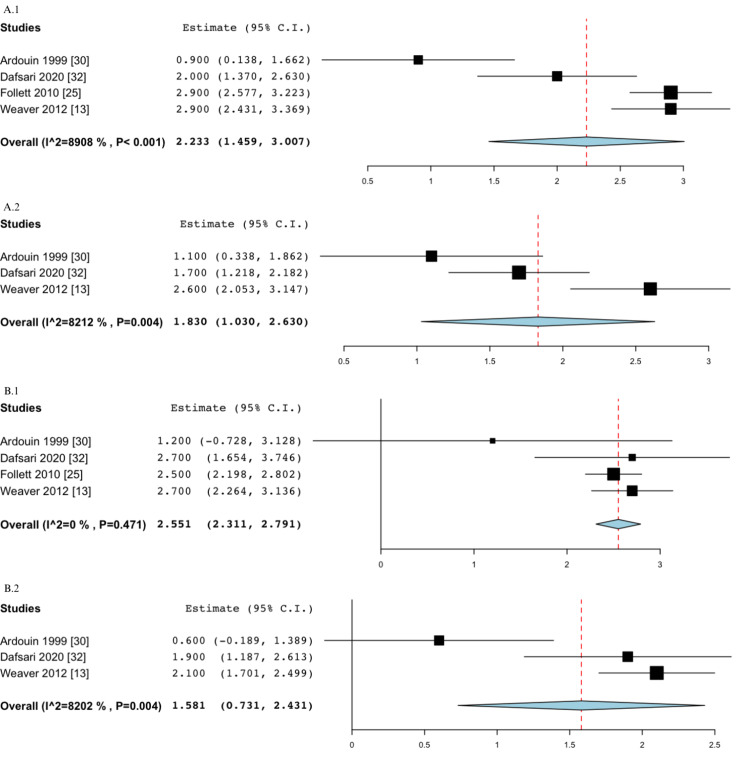
Pooled estimate of UDPRS-I outcomes between STN (A) and GPi (B) groups at baseline (A.1, B.1) and six-month follow-up (A.2, B.2) GPi: globus pallidus internus, STN: subthalamic nucleus, UPDRS: unified Parkinson's disease rating scale

Two important non-motor outcomes that have been assessed in this meta-analysis are depression and anxiety. Six studies [25-27,29,30,35] used BDI to monitor depression symptoms before and after stimulation. The mean scores at baseline were 10.65 (95% CI 8.49, 12.81, I^2^=82.43%) and 10.19 (95% CI 8.05, 12.34, I^2^=75.83%) for the STN and GPi groups, respectively. The GPi group experienced a greater score reduction at 6-12 months of follow-up with a 1.68-point decrease, while the STN group experienced a 0.84-point decrease. Neither group reached the MCID. Depression was also measured using the HADS depression-specific scale in two studies [24,28], in which the mean score decreased by 1.08 and 2.69 in the STN and GPi groups, respectively, after 12-24 months of follow-up, with the latter exceeding the MCID for improvement. Similarly, the GPi group also reached the MCID for improvement at a follow-up of 12-24 months in the HADS scale measuring anxiety [24,28] by achieving a 3.48-point decrease as opposed to a 1.36-point decrease in the STN group. Assessment of UPDRS-I at baseline in four studies [13,25,30,32] and at six months follow-up in three studies [13,30,32] revealed a decrease in 0.4 points and 0.97 points in the STN and GPi DBS groups, respectively. Results of the non-motor outcomes can be found in the Appendix (Figures 4-7).

Quality of Life

**Figure 8:**
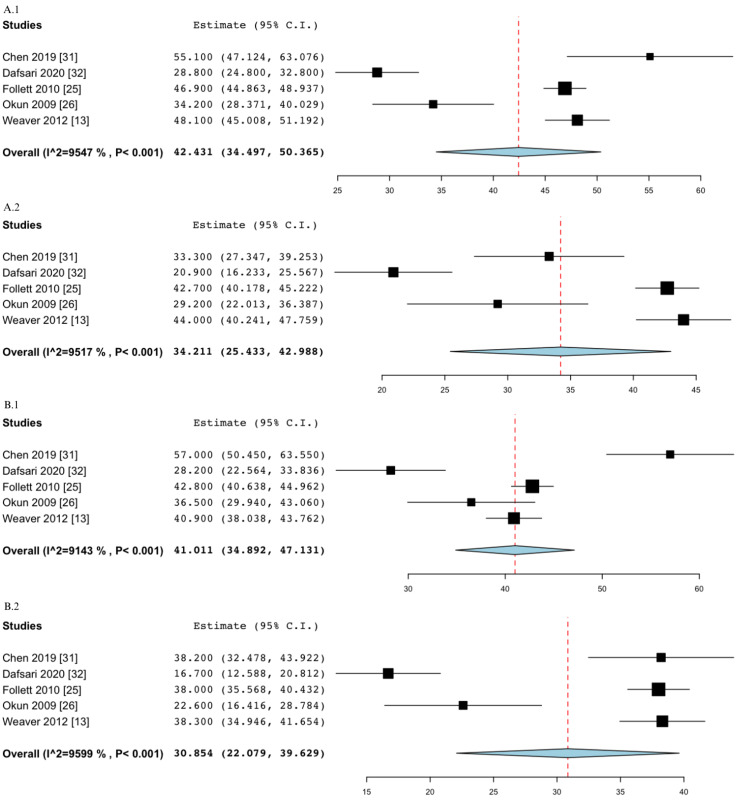
Pooled estimate of PDQ-SI outcomes between STN (A) and GPi (B) groups at baseline (A.1, B.1) and six-month follow-up (A.2, B.2) GPi: globus pallidus internus, PDQ-SI: Parkinson's disease questionnaire - summary index, STN: subthalamic nucleus

**Figure 9:**
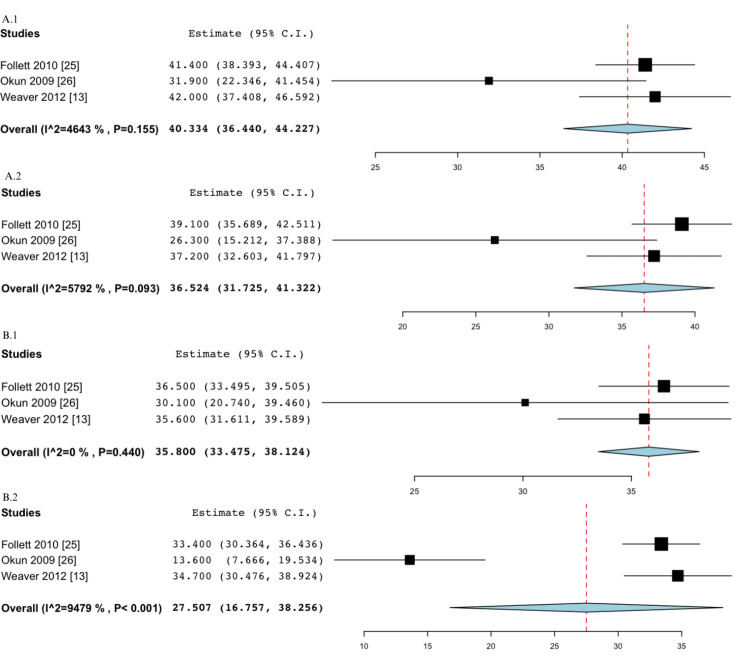
Pooled estimate of PDQ emotions outcomes between STN (A) and GPi (B) groups at baseline (A.1, B.1) and six-month follow-up (A.2, B.2) GPi: globus pallidus internus, PDQ: Parkinson's disease questionnaire, STN: subthalamic nucleus

Quality of life was assessed in five studies [13,25,26,31,32] using the PDQ-39, from which two out of the eight domains were found to be relevant to this meta-analysis. The first domain consisted of the summary index (SI), which demonstrated a greater improvement in the GPi group from baseline (mean=41.01, 95% CI 34.89, 47.13, I^2^=91.43%) to six months follow-up or more (mean=30.85, 95% CI 22.08, 39.63, I^2^=95.99%) when compared to the STN group achieving a decrease from 42.43 (95% CI 34.50, 50.37, I^2^=95.47%) at baseline to 34.21 (95% CI 25.43, 42.99, I^2^=95.17%) at follow-up. Both groups reached the MCID independently. On the other hand, an analysis of the emotions domain demonstrated that, after a follow-up of six months or greater, GPi DBS decreased PDQ-39 scores by 8.29 points compared to 3.81 points with STN DBS, with the former group achieving MCID. The above results can be found in the Appendix (Figures 8-9).

Motor Outcomes

**Figure 10:**
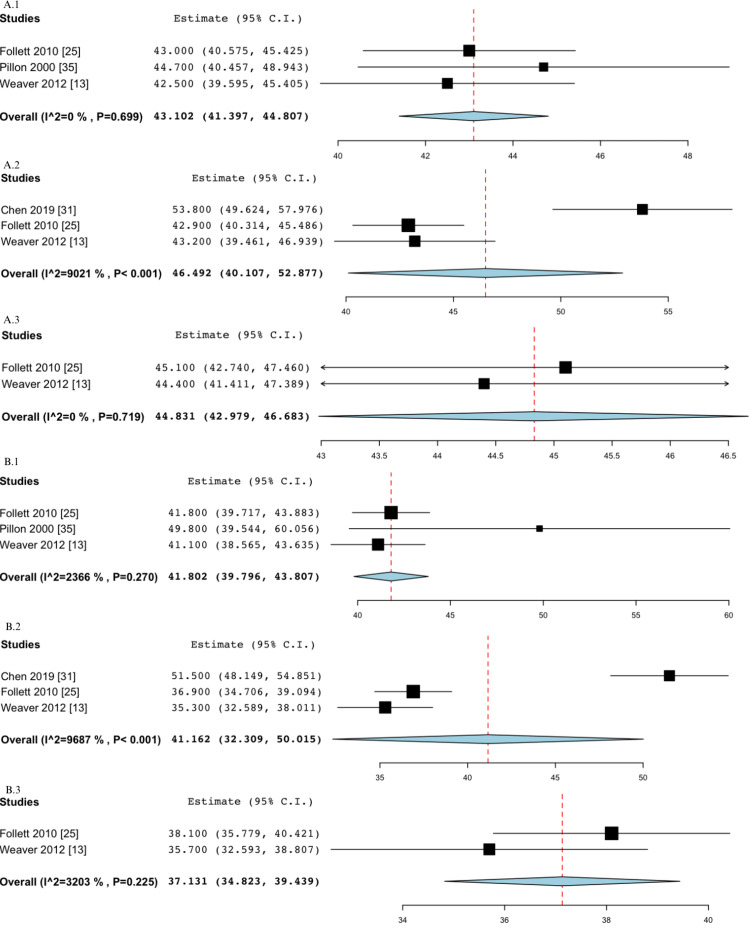
Pooled estimate of UDPRS-III off medication/off stimulation outcomes between STN (A) and GPi (B) groups at baseline (A.1, B.1), 6-12-month (A.2, B.2), and >24-month (A.3, B.3) follow-up GPi: globus pallidus internus, STN: subthalamic nucleus, UPDRS: unified Parkinson's disease rating scale

**Figure 11:**
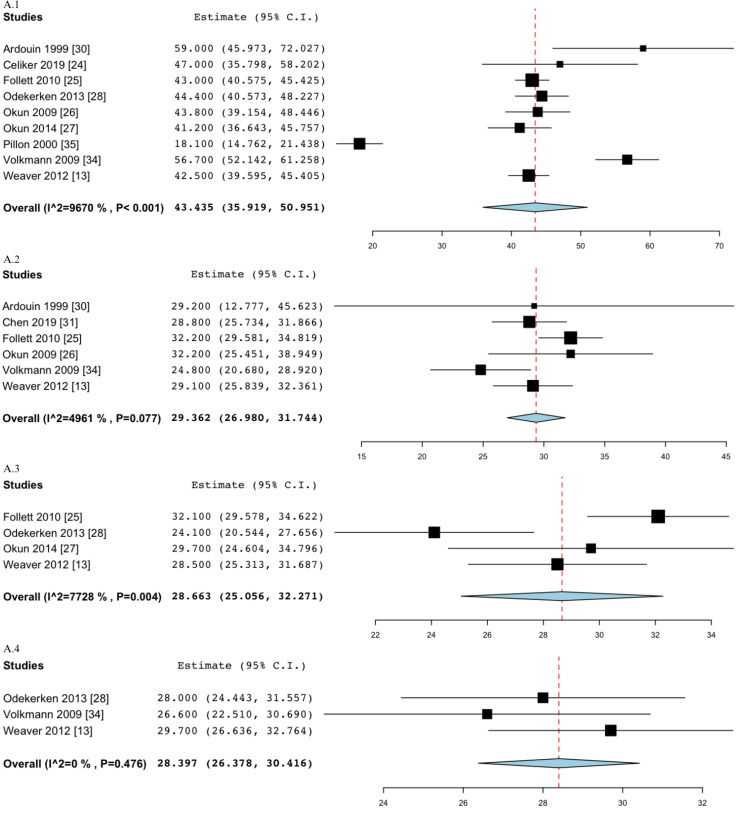
Pooled estimate of UDPRS-III off-medication/on-stimulation outcomes in the STN group at baseline (A.1), 6-12-month (A.2), 12-24-month (A.3), and >24-month (A.4) follow-up STN: subthalamic nucleus, UPDRS: unified Parkinson's disease rating scale

**Figure 12:**
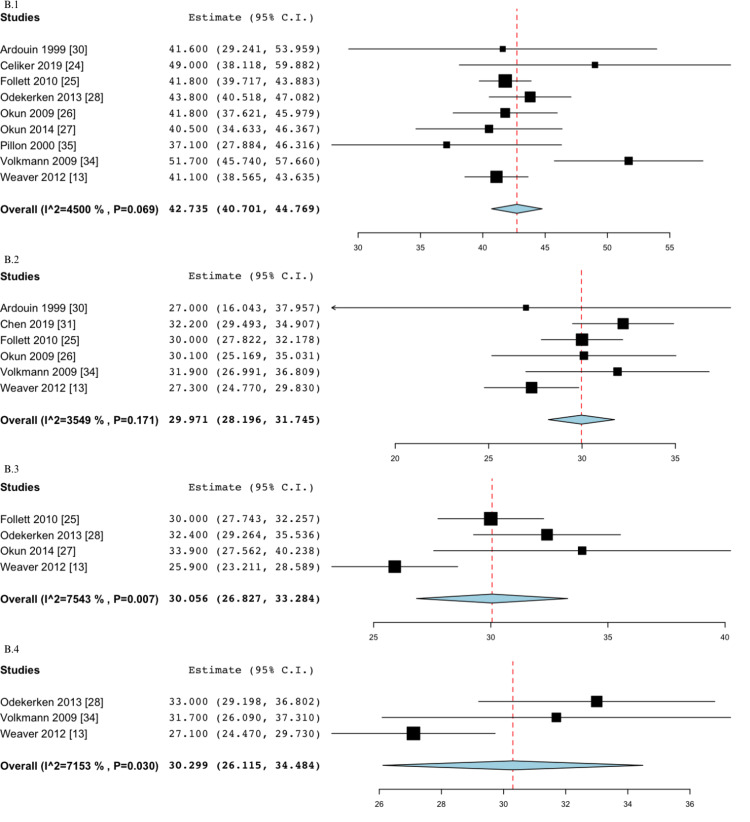
Pooled estimate of UDPRS-III off-medication/on-stimulation outcomes in the GPi group at baseline (B.1), 6-12-month (B.2), 12-24-month (B.3), and >24-month (B.4) follow-up GPi: globus pallidus internus, UPDRS: unified Parkinson's disease rating scale

**Figure 13:**
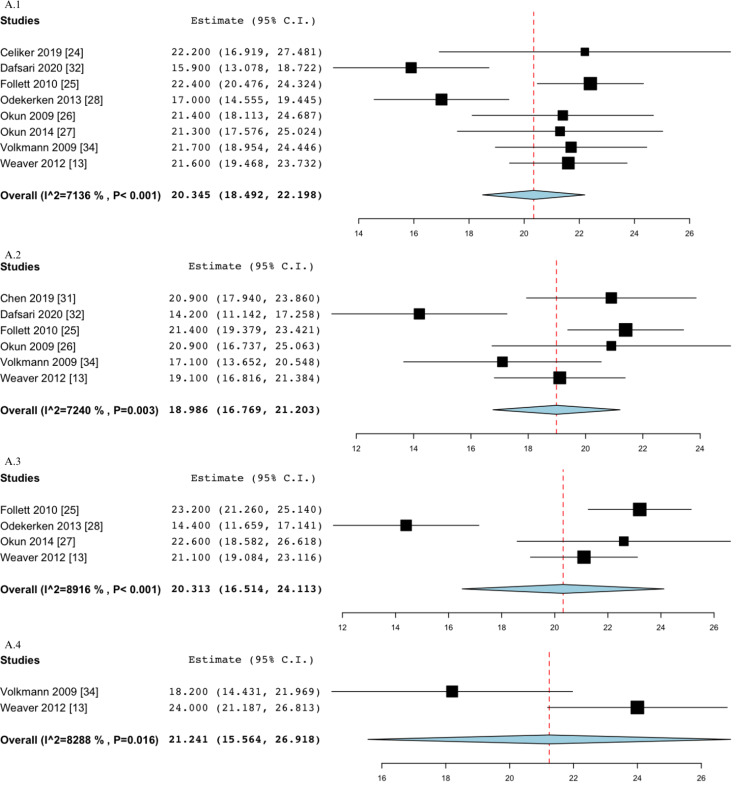
Pooled estimate of UDPRS-III on-medication/on-stimulation outcomes in the STN group at baseline (A.1), 6-12-month (A.2), 12-24-month (A.3), and >24-month (A.4) follow-up STN: subthalamic nucleus, UPDRS: unified Parkinson's disease rating scale

**Figure 14:**
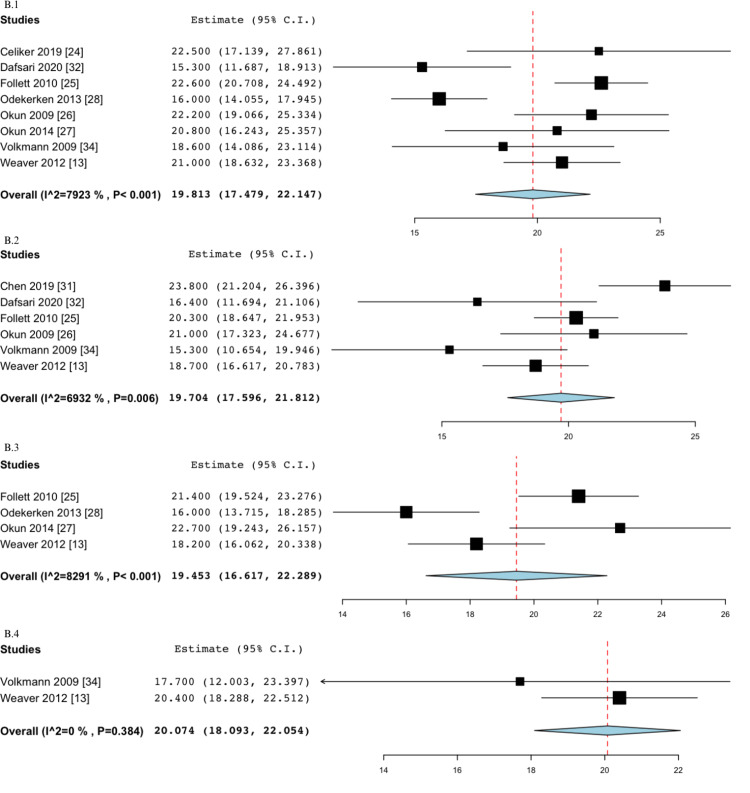
Pooled estimate of UDPRS-III on-medication/on-stimulation outcomes in the GPi group at baseline (B.1), 6-12-month (B.2), 12-24-month (B.3), and >24-month (B.4) follow-up GPi: globus pallidus internus, UPDRS: unified Parkinson's disease rating scale

Motor symptoms were assessed using the UPDRS-III scale across a combination of 11 studies [13,24-27,30-32,34,35,37]. Outcomes were measured in three different states: off medication/off stimulation, off medication/on stimulation, and on medication/on stimulation. In the off/off state, with a score change from 43.10 (95% CI 41.40, 44.81, I^2^=0%) at baseline to 46.49 (95% CI 40.11, 52.88, I^2^=90.21%) at 6-12 months and 44.83 (95% CI 42.98, 46.68, I^2^=0%) at 12-24 months in the STN group, and a decrease from 41.80 (95% CI 39.80, 43.81, I^2^=23.66%) at baseline to 41.16 (95% CI 32.31, 50.02, I^2^=96.87%) at 6-12 months and 37.13 (95% CI 34.82, 39.44, I^2^=32.03%) at 12-24 months in the GPi group, the STN group reached the MCID for symptomatic worsening at 6-12 months, while the GPi group reached the MCID for improvement at 12-24 months. In the off/on state, both groups exceeded the MCID at all follow-up periods: reductions by 14.07, 14.77, and 15.04 points in the STN group at 6-12 months, 12-24 months, and >24 months, respectively, and reductions by 12.76, 12.68, and 12.44 points in the GPi group at 6-12 months, 12-24 months, and >24 months, respectively. Finally, in the on/on state, assessment of score changes from baseline to follow-up revealed no MCID achievement. The STN group experienced a 1.36 score decrease at 6-12 months, a 0.03 score decrease at 12-24 months, and a 0.90 score increase at > 24 months, and the GPi group showed a 0.11 score decrease at 6-12 months, a 0.36 score decrease at 12-24 months, and a 0.26 score increase at > 24 months of follow-up. Results of motor outcomes can be found in the Appendix (Figures 10-14).

Levodopa Equivalent Daily Dose (LEDD)

**Figure 15:**
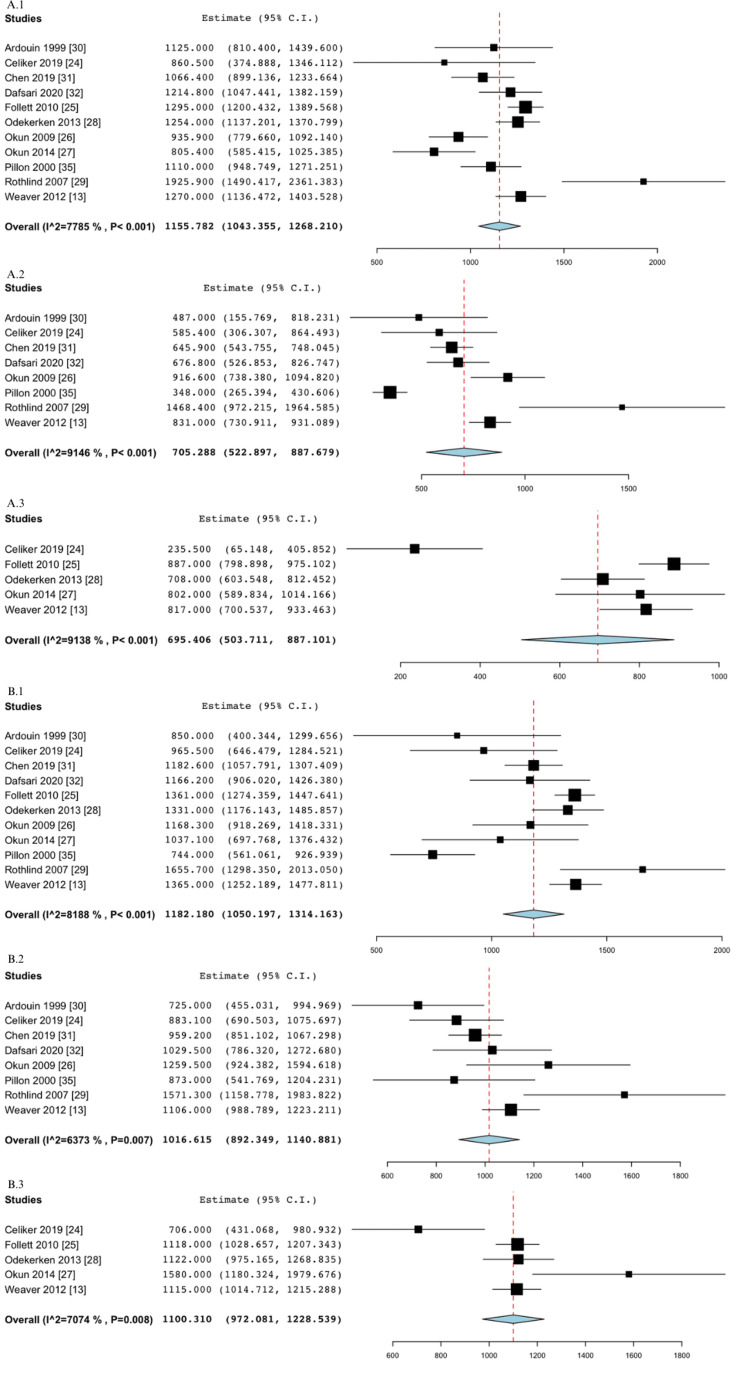
Pooled estimate of LEDD medication (mg) outcomes between STN (A) and GPi (B) groups at baseline (A.1, B.1), 6-month (A.2, B.2), and 12-month (A.3, B.3) follow-up GPi: globus pallidus internus, LEDD: levodopa equivalent daily dose, STN: subthalamic nucleus

A combination of eleven studies [13,24-32,35] assessed medication intake before and after stimulation. Baseline assessment showed an average intake of 1,155.78 mg (95% CI 1,043.136, 1,268.21, I^2^=77.85) in the STN group and 1,182.18 mg (95% CI 1,050.20, 1,314.16, I^2^=81.88%) in the GPi group. At 6-12 months of follow-up, the STN group experienced a greater reduction in medication intake than the GPi group with a 450.49 mg decrease as compared to a 165.57 mg decrease, respectively. Likewise, assessment at 12-24 months demonstrated a similar trend with a 460.38 mg reduction in the STN group as compared to an 81.87 mg reduction in the GPi group. LEDD results can be found in the Appendix (Figure 15). The results of all the above-mentioned outcomes are depicted in Table 3.

**Table 3:** Outcomes at baseline and follow-up with respective MCIDs, statistical and clinical significance Negative scores signify improvement, positive scores signify worsening. † Follow-up for BDI and PDQ-39 (SI and emotions) was for > 6 months ‡ Follow-up for HADS was done at 12 months †† Follow-up for LEDD was done at 6 months and at > 12 months ‡‡ STN group reached MCID at 6-12 months = clinically meaningful worsening, GPi group reached MCID at 12-24 months = clinically meaningful improvement § STN and GPi groups reached MCID at all time points = clinically meaningful improvement ¶ GPi group reached MCID at 12-24 months = clinically meaningful improvement §§ STN and GPi groups reached MCID at 6-12 months = clinically meaningful improvement ¶¶ GPi group reached MCID at 6-12 months = clinically meaningful improvement BDI: Beck depression inventory, GPi: globus pallidus internus, HADS: hospital anxiety and depression scale, LEDD: levodopa equivalent daily dose, MCID: minimum clinically important difference, N: number of studies, N/A: not applicable, NR: not reported, PDQ: Parkinson’s Disease questionnaire, SD: standard deviation, SI: summary index, STN: subthalamic nucleus, UPDRS: unified Parkinson’s disease rating scale

Outcome	MCID	Mean (N)								Change from baseline = follow-up mean – baseline mean						Significance
		Baseline		6-12 months		12-24 months		> 24 months		6-12 months		12-24 months		> 24 months		
		STN	GPi	STN	GPi	STN	GPi	STN	GPi	STN	GPi	STN	GPi	STN	GPi	
UDPRS-I	N/A	2.23 (4)	2.55 (4)	1.83 (3)	1.58 (3)	NR	NR	NR	NR	-0.40	-0.97	NR	NR	NR	NR	N/A
UPDRS-III (off/off)	3.25	43.10 (3)	41.80 (3)	46.49 (3)	41.16 (3)	44.83 (2)	37.13 (2)	NR	NR	3.39	-0.64	1.73	-4.67	NR	NR	‡‡
UPDRS-III (off/on)	3.25	43.44 (9)	42.74 (9)	29.36 (6)	29.97 (6)	28.66 (4)	30.06 (4)	28.40 (3)	30.30 (3)	-14.07	-12.76	-14.77	-12.68	-15.04	-12.44	§
UPDRS-III (on/on)	3.25	20.35 (8)	19.81 (8)	18.99 (6)	19.70 (6)	20.31 (4)	19.45 (4)	21.24 (2)	20.07 (2)	-1.36	-0.11	-0.03	-0.36	0.90	0.26	None
BDI †	3.30	10.65 (6)	10.19 (6)	9.81 (6)	8.51 (6)	NR	NR	NR	NR	-0.84	-1.68	NR	NR	NR	NR	None
HADS (depression) ‡	1.72	7.34 (2)	8.76 (2)	NR	NR	6.26 (2)	6.07 (2)	NR	NR	NR	NR	-1.08	-2.69	NR	NR	¶
HADS (anxiety) ‡	1.84	7.38 (2)	9.43 (2)	NR	NR	6.02 (2)	5.96 (2)	NR	NR	NR	NR	-1.36	-3.48	NR	NR	¶
PDQ-39 (SI) †	-4.72 (improvement) 4.22 (worsening)	42.43 (5)	41.01 (5)	34.21 (5)	30.85 (5)	NR	NR	NR	NR	-8.22	-10.16	NR	NR	NR	NR	§§
PDQ-39 (emotions) †	-4.72 (improvement) 4.22 (worsening)	40.33 (3)	35.80 (3)	36.52 (3)	27.51 (3)	NR	NR	NR	NR	-3.81	-8.29	NR	NR	NR	NR	¶¶
LEDD (mg) ††	N/A	1155.78 (11)	1182.18 (11)	705.29 (8)	1016.62 (8)	695.41 (5)	1100.31 (5)	NR	NR	-450.49	-165.57	-460.38	-81.87	NR	NR	N/A

Discussion

To the best of our knowledge, this meta-analysis is the first to evaluate the MCID in depression, anxiety, and quality of life between PD patients with STN-DBS and GPi-DBS. While simultaneously assessing those outcomes against motor symptoms and medication change, our study attempted to establish whether either target could be prioritized based on patient outcomes in PD.

Our study demonstrated that, after a minimum of six months of follow-up, GPi stimulation showed a greater improvement in depressive symptoms when compared to STN stimulation, but as neither group reached the MCID, the score change on BDI was not clinically meaningful. However, HADS assessment showed that the GPi group achieved a clinically meaningful improvement in depression symptoms after 12 months and a greater score reduction in the UPDRS-I after six months when compared to the STN group. The lesser improvements in depression after STN DBS could be explained by the STN’s smaller volume and close proximity to limbic and other non-motor fiber pathways [29] whose risk of stimulation increases when the STN is stimulated more anteriorly and ventrally [38]. For instance, networks responsible for mood, thinking, and reward such as the anterior cingulate cortex, orbitofrontal cortex, and ventral tegmental area have a higher chance of receiving diffused current when electrodes are placed closer to the ventromedial STN. With an overlap of functional areas (motor, cognitive, and emotional) in the STN, hypomanic manifestations are often encountered after its stimulation medially due to a strong interaction between motor and non-motor regions and inhibition of excitatory projections by adjacent current [39]. Thus, shifting stimulation to target a more dorsal region within the STN is beneficial in reducing some neuropsychiatric symptoms [38]. In contrast, the GPi’s motor region volume is relatively larger than that of the STN and allows for a decreased chance of stimulating current reaching non-motor areas and producing non-motor adverse effects [38]. However, by applying adaptive and targeted stimulation based on recorded neurophysiological activity, intermittent DBS could then be delivered and tailored to the patient’s needs to produce the desired effects, rather than having non-motor areas being randomly affected by adjacent currents [39]. In addition, due to variable patterns of cell loss in brains affected by PD and differing rates of motor and non-motor pathway degeneration, the dopaminergic projections in those pathways may respond differently to a given stimulation level, which might cause a functional imbalance in an adjacent non degenerated region responsible for cognitive and limbic circuits [40]. Moreover, pseudobulbar or cognitive deficits may arise with bilaterally placed electrodes that run the risk of irritating non-motor areas [41]; thus, unilateral or bilateral electrode implantation might differently modulate the limbic and extra-limbic systems that play a role in mood regulation. Nevertheless, the selection of the GPi as a target for stimulation may provide an advantageous effect on alleviating depressive symptoms, reducing their occurrence, and improving quality of life [6,42].

Our study showed that patients undergoing GPi stimulation experienced a clinically meaningful reduction in anxiety after 12 months as measured by the HADS anxiety-specific scale. On the other hand, a previous meta-analysis investigating this outcome using the state-trait anxiety inventory (STAI) and summarizing the results with the standardized mean difference (SMD) found no difference between the two treatments [43]. Those findings could suggest that the GPi might be a more favorable target in PD treatment if one were to value reductions in anxiety, and this could also be explained by what has been previously mentioned about the STN’s effect on depression. Likewise, ventral STN stimulation has been reported to induce hypomanic states with symptoms of hyperactivity, decreased need for sleep, or reward-seeking behavior potentially disrupting a patient’s relationships [38]. However, with only two studies assessing the effects of DBS on anxiety, our results necessitate stronger evidence for increased certainty and reliability.

This meta-analysis also assessed motor symptoms against non-motor ones to establish whether any outcome or target could be prioritized for optimal patient care delivery and long-term disability reduction. UPDRS-III motor scores were evaluated under three states - off/off, off/on, and on/on medication and stimulation - and according to previous publications, UPDRS-III score changes in the off medication/on stimulation state are most commonly used to measure DBS efficacy [44]. Hence, despite both treatment groups achieving clinically meaningful improvements in motor symptoms at all follow-up periods, our findings suggest a potential favorability toward the STN since its stimulation demonstrated greater UPDRS-III score reductions. Those results were also found to be in agreement with a recently published meta-analysis [44]. STN stimulation has been shown by several studies to reduce dyskinesia since it is superior to GPi stimulation in reducing the levodopa equivalent daily dose and thus reducing levodopa-induced dyskinesia [44]. Therefore, as also shown by our results, GPi stimulation might not be as advantageous as STN stimulation in improving motor symptoms and alleviating dyskinesias. However, given that this score does not take into consideration non-motor symptoms, it is yet unlikely to establish the superiority of either target in that regard. This necessitates the use of an assessment tool that takes motor and non-motor aspects into account; thus, the importance of quality of life measured by PDQ-39 emerges and seems to be a more appropriate assessment tool for DBS efficacy [44] and the selection of a surgical target.

Quality-of-life outcomes showed greater score reductions after six months of GPi stimulation; while both groups achieved a clinically meaningful improvement in the summary index domain of the PDQ-39, the effects of GPi stimulation seemed to be more favorable, as shown by a score reduction exceeding the MCID in the emotions domain. Our findings are in line with a previous meta-analysis [45], showing improvement in nearly all quality-of-life domains after GPi DBS as compared to STN DBS. Such results might present an advantageous effect in opting for pallidal stimulation when clinicians decide to prioritize their patients’ quality of life. PDQ-39 could be considered to reveal the overall outcomes of DBS, as its perception relies on motor [46] and non-motor symptom improvement, which both contribute significantly to quality of life [45]. However, it should be stressed again that motor symptoms should not be disregarded as improvement in this domain could provide relief in patients, contribute to fewer feelings of anxiety [11], and ultimately improve overall quality of life [46].

This study also aimed to investigate the effects of stimulation on medication change. As shown by our results, and as also indicated by several previous meta-analyses [45,47], a greater reduction in levodopa was observed in the STN group after a follow-up of six months and onwards, as opposed to the GPi group. Those findings might provide an advantage to patients suffering from poor compliance and from levodopa-induced dyskinesia due to chronic medication intake [45]. Although a greater medication reduction might improve quality of life, there could be an increased risk of postoperative dopamine withdrawal symptoms as the central nervous system readjusts to a new state after STN DBS and direct levodopa interruption [11]. Nevertheless, other studies have shown reduced rates of levodopa-induced dyskinesia and improved postoperative activities of daily living following GPi stimulation [48]. While GPi stimulation, which seems to be associated with higher postoperative LEDD [45], could offer an advantage in helping to reduce non-motor side effects [40,49] and still control dyskinesias [48], STN stimulation offers the benefit of greater medication reductions. Hence, choosing a target according to those findings would be based on what patients value more - a greater medication reduction for those suffering from dyskinesias versus reduced dopamine withdrawal symptoms.

There were several limitations to this study. Firstly, since the STN has widely been adopted as a surgical approach [27] and is used more commonly for the treatment of PD [25], there exists more data about its motor and non-motor side effects, as compared to the GPi [32]. More randomized controlled trials are needed to explore the effects of GPi DBS on non-motor outcomes and to compare them to those of STN DBS. This might enable researchers and clinicians to provide a general picture of a preferred stimulation location for PD patients. In addition, variabilities in baseline and clinical characteristics, such as differences in disease severity and duration, might affect certainty in results. Furthermore, there seems to be an inconsistency in measurement methods, procedures, and postoperative assessments, which could increase the study’s heterogeneity. For instance, surgeries were performed with various implantation techniques targeting different locations within each STN and GPi. Patients were assessed under different states - on versus off medication, on versus off stimulation - and were subject to different stimulation parameters, such as voltage, frequency, and laterality of electrode implantation. Postoperative assessments were also conducted using different scales (BDI or HADS for depression evaluation) and at various follow-up durations. Another major limitation was the inability to assess and control for biases within individual studies. Lastly, to our knowledge, MCID for LEDD has not been reported in the literature, which made it difficult to assess clinically meaningful differences.

## Conclusions

This study demonstrated that, although the STN provided greater medication reductions and motor improvement post-operatively, GPi stimulation seemed to have a positive impact on mood and quality of life. While clinicians should be aware of the conflicting evidence around optimal stimulation location, this study might help them guide patients throughout their DBS options while simultaneously prioritizing a patient’s subjective performance, personal preferences, clinical motor and non-motor responses, and quality of life. Therefore, this study hopes to provide an opportunity for further research into this subject matter and an insight into future clinical practice.

## Appendices

**Table 4:** Search strategies

Database	Searches
OVID Medline	*Deep Brain Stimulation/ or *"Transcranial Magnetic Stimulation"/ or *"Transcranial Direct Current Stimulation"/ or exp *"noninvasive brain stimulation"/ or exp *"brain depth stimulation"/ or *"transcranial electrical stimulation"/ (((stimulation or "electro-stimulat*" or electrostim* or "electro-therap*" or electrotherap*) adj2 ("Nucleus Accumbens" or "Subthalamic Nucleus" or brain or cortex or cortical or transcranial or cranial)) or tDCS or (deep adj (rTMS or TMS))).ti. (fatigue or mood* or sleep* or wakeful* or cognition or cognitive or apathy or fatigue or depressive or depression or mania or neurobehavioural or neurobehavioral or psychiatric or neuropsychological or personality or appetite or weight gain or cardiovascular or gastrointestinal).ti. or (nonmotor or "non-motor").ti,ab,hw,kw. or nonmotor symptom/ *Fatigue/ *Mania/ *Depression/ *Depressive Disorder/ *Sleep/ Wakefulness/ or daytime somnolence/ or "Disorders of Excessive Somnolence"/ or Sleep Wake Disorders/ Weight Gain/ or body weight gain/ *Apathy/ *Personality/ *Personality Disorders/ or personality disorder/ *Appetite/ exp *Cardiovascular Physiological Phenomena/ or *cardiovascular function/ exp *Digestive System Physiological Phenomena/ *Memory/ exp *Cognition/ or *Executive Function/ exp *Mood Disorders/ or *Mental Disorders/ or *Parkinson Disease/ (parkinson* or PD).ti.(exp animals/ or exp nonhuman/) not exp humans/ ((alpaca or alpacas or amphibian or amphibians or animal or animals or antelope or armadillo or armadillos or avian or baboon or baboons or beagle or beagles or bee or bees or bird or birds or bison or bovine or buffalo or buffaloes or buffalos or "c elegans" or "Caenorhabditis elegans" or camel or camels or canine or canines or carp or cats or cattle or chick or chicken or chickens or chicks or chimp or chimpanze or chimpanzees or chimps or cow or cows or "D melanogaster" or "dairy calf" or "dairy calves" or deer or dog or dogs or donkey or donkeys or drosophila or "Drosophila melanogaster" or duck or duckling or ducklings or ducks or equid or equids or equine or equines or feline or felines or ferret or ferrets or finch or finches or fish or flatworm or flatworms or fox or foxes or frog or frogs or "fruit flies" or "fruit fly" or "G mellonella" or "Galleria mellonella" or geese or gerbil or gerbils or goat or goats or goose or gorilla or gorillas or hamster or hamsters or hare or hares or heifer or heifers or horse or horses or insect or insects or jellyfish or kangaroo or kangaroos or kitten or kittens or lagomorph or lagomorphs or lamb or lambs or llama or llamas or macaque or macaques or macaw or macaws or marmoset or marmosets or mice or minipig or minipigs or mink or minks or monkey or monkeys or mouse or mule or mules or nematode or nematodes or octopus or octopuses or orangutan or "orang-utan" or orangutans or "orang-utans" or oxen or parrot or parrots or pig or pigeon or pigeons or piglet or piglets or pigs or porcine or primate or primates or quail or rabbit or rabbits or rat or rats or reptile or reptiles or rodent or rodents or ruminant or ruminants or salmon or sheep or shrimp or slug or slugs or swine or tamarin or tamarins or toad or toads or trout or urchin or urchins or vole or voles or waxworm or waxworms or worm or worms or xenopus or "zebra fish" or zebrafish) not (human or humans or patient or patients)).ti,ab,hw,kw. (rat or rats or mice or mouse or murine or pig or pigs or porcine or swine or dog or dogs).ti. or/ (conference abstract or conference review or editorial or erratum or note or addresses or autobiography or bibliography or biography or blogs or comment or dictionary or directory or interactive tutorial or interview or lectures or legal cases or legislation or news or newspaper article or patient education handout or periodical index or portraits or published erratum or video-audio media or webcasts).mp. or conference abstract.st. limit to english language
Cochrane Library	MeSH descriptor: [Deep Brain Stimulation] explode all trees MeSH descriptor: [Transcranial Magnetic Stimulation] explode all trees MeSH descriptor: [Transcranial Direct Current Stimulation] explode all trees MeSH descriptor: [Transcranial Direct Current Stimulation] explode all trees (((stimulation OR 'electro-stimulat*' OR electrostim* OR 'electro-therap*' OR electrotherap*) NEAR/2 ('nucleus accumbens' OR 'subthalamic nucleus' OR brain OR cortex OR cortical OR transcranial OR cranial))):ti (Word variations have been searched) (tdcs OR (deep NEAR/2 (rtms OR tms))):ti (Word variations have been searched) depressive or depression or mania or neurobehavioural or neurobehavioral or psychiatric or neuropsychological or personality or appetite or "weight gain" or cardiovascular or gastrointestinal):ti (Word variations have been searched) (nonmotor or "non-motor"):ti,ab,kw (Word variations have been searched) ("nonmotor symptom"):ti,ab,kw (Word variations have been searched) MeSH descriptor: [Fatigue] this term only MeSH descriptor: [Mania] this term only MeSH descriptor: [Depression] this term only MeSH descriptor: [Depressive Disorder] this term only MeSH descriptor: [Sleep] this term only MeSH descriptor: [Wakefulness] explode all trees MeSH descriptor: [Disorders of Excessive Somnolence] explode all trees MeSH descriptor: [Sleep Wake Disorders] explode all trees MeSH descriptor: [Weight Gain] explode all trees MeSH descriptor: [Apathy] this term only MeSH descriptor: [Personality] this term only MeSH descriptor: [Personality Disorders] this term only MeSH descriptor: [Appetite] this term only MeSH descriptor: [Cardiovascular Physiological Phenomena] explode all trees MeSH descriptor: [Digestive System Physiological Phenomena] explode all trees MeSH descriptor: [undefined] explode all trees MeSH descriptor: [Cognition] explode all trees MeSH descriptor: [Executive Function] explode all trees MeSH descriptor: [Parkinson Disease] this term only (parkinson* OR PD):ti (Word variations have been searched) MeSH descriptor: [Humans] explode all trees MeSH descriptor: [Animals] explode all trees (((alpaca or alpacas or amphibian or amphibians or animal or animals or antelope or armadillo or armadillos or avian or baboon or baboons or beagle or beagles or bee or bees or bird or birds or bison or bovine or buffalo or buffaloes or buffalos or "c elegans" or "Caenorhabditis elegans" or camel or camels or canine or canines or carp or cats or cattle or chick or chicken or chickens or chicks or chimp or chimpanze or chimpanzees or chimps or cow or cows or "D melanogaster" or "dairy calf" or "dairy calves" or deer or dog or dogs or donkey or donkeys or drosophila or "Drosophila melanogaster" or duck or duckling or ducklings or ducks or equid or equids or equine or equines or feline or felines or ferret or ferrets or finch or finches or fish or flatworm or flatworms or fox or foxes or frog or frogs or "fruit flies" or "fruit fly" or "G mellonella" or "Galleria mellonella" or geese or gerbil or gerbils or goat or goats or goose or gorilla or gorillas or hamster or hamsters or hare or hares or heifer or heifers or horse or horses or insect or insects or jellyfish or kangaroo or kangaroos or kitten or kittens or lagomorph or lagomorphs or lamb or lambs or llama or llamas or macaque or macaques or macaw or macaws or marmoset or marmosets or mice or minipig or minipigs or mink or minks or monkey or monkeys or mouse or mule or mules or nematode or nematodes or octopus or octopuses or orangutan or "orang-utan" or orangutans or "orang-utans" or oxen or parrot or parrots or pig or pigeon or pigeons or piglet or piglets or pigs or porcine or primate or primates or quail or rabbit or rabbits or rat or rats or reptile or reptiles or rodent or rodents or ruminant or ruminants or salmon or sheep or shrimp or slug or slugs or swine or tamarin or tamarins or toad or toads or trout or urchin or urchins or vole or voles or waxworm or waxworms or worm or worms or xenopus or "zebra fish" or zebrafish) not (human or humans or patient or patients))):ti,ab,kw (Word variations have been searched) ((rat or rats or mice or mouse or murine or pig or pigs or porcine or swine or dog or dogs)):ti (Word variations have been searched)
PsycInfo via APA PsycNet	((((AnyField:("Deep Brain Stimulation"))) OR ((AnyField:("Transcranial Magnetic Stimulation"))) OR ((AnyField:("Transcranial Direct Current Stimulation"))) OR ((AnyField:("noninvasive brain stimulation"))) OR ((AnyField:("brain depth stimulation"))) OR ((AnyField:("transcranial electrical stimulation")))) OR ((((title:(stimulation)) OR (title:('electro-stimulat*')) OR (title:(electrostim*)) OR (title:('electro-therap*')) OR (title:(electrotherap*))) NEAR/2 ((title:('nucleus accumbens')) OR (title:('subthalamic nucleus')) OR (title:(brain)) OR (title:(cortex)) OR (title:(cortical)) OR (title:(transcranial)) OR (title:(cranial))))) OR (((Title:(tDCS) OR (Title:(deep NEAR/2) (Title:(rTMS) OR Title:(TMS))))))) AND ((((MeSH: (Fatigue)))) OR (((MeSH: (Mania))) OR ((MeSH: (Depression)))) OR (((MeSH: (depressive disorder)))) OR (((MeSH: (Sleep)))) OR (((Any Field: (Wakefulness)) OR (Any Field: ("daytime somnolence")) OR (Any Field: ("Disorders of Excessive Somnolence")) OR (Any Field: (Sleep Wake Disorders)))) OR (((Any Field: ("Weight Gain")) OR (Any Field: ("body weight gain")))) OR (((MeSH: (apathy))) OR ((MeSH: (personality))) OR ((MeSH: (appetite)))) OR (((MeSH: (Cardiovascular Physiological Phenomena)))) OR (((MeSH: (cardiovascular function)))) OR (((MeSH: (Digestive System Physiological Phenomena)))) OR (((MeSH: (Memory))) OR ((MeSH: (cognition))) OR ((MeSH: (executive function)))) OR (((MeSH: (mood disorders))) OR ((MeSH: (mental disorders)))) OR (((MeSH: (personality disorders))))) AND ((((title: (parkinson*))) OR ((title: (PD)))) OR (((MeSH: (parkinson disease)))))
Embase	rat:ti OR rats:ti OR mice:ti OR mouse:ti OR murine:ti OR pig:ti OR pigs:ti OR porcine:ti OR swine:ti OR dog:ti OR dogs:ti (alpaca:ti,ab,kw OR alpacas:ti,ab,kw OR amphibian:ti,ab,kw OR amphibians:ti,ab,kw OR animal:ti,ab,kw OR animals:ti,ab,kw OR antelope:ti,ab,kw OR armadillo:ti,ab,kw OR armadillos:ti,ab,kw OR avian:ti,ab,kw OR baboon:ti,ab,kw OR baboons:ti,ab,kw OR beagle:ti,ab,kw OR beagles:ti,ab,kw OR bee:ti,ab,kw OR bees:ti,ab,kw OR bird:ti,ab,kw OR birds:ti,ab,kw OR bison:ti,ab,kw OR bovine:ti,ab,kw OR buffalo:ti,ab,kw OR buffaloes:ti,ab,kw OR buffalos:ti,ab,kw OR 'c elegans':ti,ab,kw OR 'caenorhabditis elegans':ti,ab,kw OR camel:ti,ab,kw OR camels:ti,ab,kw OR canine:ti,ab,kw OR canines:ti,ab,kw OR carp:ti,ab,kw OR cats:ti,ab,kw OR cattle:ti,ab,kw OR chick:ti,ab,kw OR chicken:ti,ab,kw OR chickens:ti,ab,kw OR chicks:ti,ab,kw OR chimp:ti,ab,kw OR chimpanze:ti,ab,kw OR chimpanzees:ti,ab,kw OR chimps:ti,ab,kw OR cow:ti,ab,kw OR cows:ti,ab,kw OR 'd melanogaster':ti,ab,kw OR 'dairy calf':ti,ab,kw OR 'dairy calves':ti,ab,kw OR deer:ti,ab,kw OR dog:ti,ab,kw OR dogs:ti,ab,kw OR donkey:ti,ab,kw OR donkeys:ti,ab,kw OR drosophila:ti,ab,kw OR 'drosophila melanogaster':ti,ab,kw OR duck:ti,ab,kw OR duckling:ti,ab,kw OR ducklings:ti,ab,kw OR ducks:ti,ab,kw OR equid:ti,ab,kw OR equids:ti,ab,kw OR equine:ti,ab,kw OR equines:ti,ab,kw OR feline:ti,ab,kw OR felines:ti,ab,kw OR ferret:ti,ab,kw OR ferrets:ti,ab,kw OR finch:ti,ab,kw OR finches:ti,ab,kw OR fish:ti,ab,kw OR flatworm:ti,ab,kw OR flatworms:ti,ab,kw OR fox:ti,ab,kw OR foxes:ti,ab,kw OR frog:ti,ab,kw OR frogs:ti,ab,kw OR 'fruit flies':ti,ab,kw OR 'fruit fly':ti,ab,kw OR 'g mellonella':ti,ab,kw OR 'galleria mellonella':ti,ab,kw OR geese:ti,ab,kw OR gerbil:ti,ab,kw OR gerbils:ti,ab,kw OR goat:ti,ab,kw OR goats:ti,ab,kw OR goose:ti,ab,kw OR gorilla:ti,ab,kw OR gorillas:ti,ab,kw OR hamster:ti,ab,kw OR hamsters:ti,ab,kw OR hare:ti,ab,kw OR hares:ti,ab,kw OR heifer:ti,ab,kw OR heifers:ti,ab,kw OR horse:ti,ab,kw OR horses:ti,ab,kw OR insect:ti,ab,kw OR insects:ti,ab,kw OR jellyfish:ti,ab,kw OR kangaroo:ti,ab,kw OR kangaroos:ti,ab,kw OR kitten:ti,ab,kw OR kittens:ti,ab,kw OR lagomorph:ti,ab,kw OR lagomorphs:ti,ab,kw OR lamb:ti,ab,kw OR lambs:ti,ab,kw OR llama:ti,ab,kw OR llamas:ti,ab,kw OR macaque:ti,ab,kw OR macaques:ti,ab,kw OR macaw:ti,ab,kw OR macaws:ti,ab,kw OR marmoset:ti,ab,kw OR marmosets:ti,ab,kw OR mice:ti,ab,kw OR minipig:ti,ab,kw OR minipigs:ti,ab,kw OR mink:ti,ab,kw OR minks:ti,ab,kw OR monkey:ti,ab,kw OR monkeys:ti,ab,kw OR mouse:ti,ab,kw OR mule:ti,ab,kw OR mules:ti,ab,kw OR nematode:ti,ab,kw OR nematodes:ti,ab,kw OR octopus:ti,ab,kw OR octopuses:ti,ab,kw OR orangutan:ti,ab,kw OR 'orang-utan':ti,ab,kw OR orangutans:ti,ab,kw OR 'orang-utans':ti,ab,kw OR oxen:ti,ab,kw OR parrot:ti,ab,kw OR parrots:ti,ab,kw OR pig:ti,ab,kw OR pigeon:ti,ab,kw OR pigeons:ti,ab,kw OR piglet:ti,ab,kw OR piglets:ti,ab,kw OR pigs:ti,ab,kw OR porcine:ti,ab,kw OR primate:ti,ab,kw OR primates:ti,ab,kw OR quail:ti,ab,kw OR rabbit:ti,ab,kw OR rabbits:ti,ab,kw OR rat:ti,ab,kw OR rats:ti,ab,kw OR reptile:ti,ab,kw OR reptiles:ti,ab,kw OR rodent:ti,ab,kw OR rodents:ti,ab,kw OR ruminant:ti,ab,kw OR ruminants:ti,ab,kw OR salmon:ti,ab,kw OR sheep:ti,ab,kw OR shrimp:ti,ab,kw OR slug:ti,ab,kw OR slugs:ti,ab,kw OR swine:ti,ab,kw OR tamarin:ti,ab,kw OR tamarins:ti,ab,kw OR toad:ti,ab,kw OR toads:ti,ab,kw OR trout:ti,ab,kw OR urchin:ti,ab,kw OR urchins:ti,ab,kw OR vole:ti,ab,kw OR voles:ti,ab,kw OR waxworm:ti,ab,kw OR waxworms:ti,ab,kw OR worm:ti,ab,kw OR worms:ti,ab,kw OR xenopus:ti,ab,kw OR 'zebra fish':ti,ab,kw OR zebrafish:ti,ab,kw) NOT (human:ti,ab,kw OR humans:ti,ab,kw OR patient:ti,ab,kw OR patients:ti,ab,kw) 'mental disorders'/mj 'mood disorder'/exp OR 'mood disorder' 'executive function'/mj 'cognition'/exp OR cognition 'memory'/mj 'digestive system physiological phenomena'/exp OR 'digestive system physiological phenomena' 'cardiovascular physiological phenomena'/exp OR 'cardiovascular physiological phenomena' 'cardiovascular function'/exp OR 'cardiovascular function' 'appetite'/mj 'personality disorders'/mj 'personality disorder'/mj 'personality'/mj 'apathy'/mj 'weight gain'/exp OR 'weight gain' 'body weight gain'/exp OR 'body weight gain' 'sleep wake disorders'/exp OR 'sleep wake disorders' 'disorders of excessive somnolence'/exp OR 'disorders of excessive somnolence' 'daytime somnolence'/exp OR 'daytime somnolence' 'wakefulness'/exp OR wakefulness 'sleep'/mj 'depressive disorder'/mj 'depression'/mj 'mania'/mj 'fatigue'/mj 'nonmotor symptom'/exp OR 'nonmotor symptom' mood*:ti,ab,kw OR sleep*:ti,ab,kw OR wakeful*:ti,ab,kw OR cognition:ti,ab,kw OR cognitive:ti,ab,kw OR apathy:ti,ab,kw OR fatigue:ti,ab,kw OR depressive:ti,ab,kw OR depression:ti,ab,kw OR mania:ti,ab,kw OR neurobehavioural:ti,ab,kw OR neurobehavioral:ti,ab,kw OR psychiatric:ti,ab,kw OR neuropsychological:ti,ab,kw OR personality:ti,ab,kw OR appetite:ti,ab,kw OR 'weight gain':ti,ab,kw OR cardiovascular:ti,ab,kw OR gastrointestinal:ti,ab,kw (((stimulation OR 'electro-stimulat*' OR electrostim* OR 'electro-therap*' OR electrotherap*) NEAR/2 ('nucleus accumbens' OR 'subthalamic nucleus' OR brain OR cortex OR cortical OR transcranial OR cranial)):ti) OR tdcs:ti OR ((deep NEAR/2 (rtms OR tms)):ti 'transcranial electrical stimulation'/'brain depth stimulation'/exp OR 'brain depth stimulation' 'noninvasive brain stimulation'/exp OR 'noninvasive brain 'transcranial direct current stimulation'/ 'transcranial magnetic stimulation'/mj 'deep brain stimulation'/mj
Scopus	TITLE (((stimulation or "electro-stimulat*" or electrostim* or "electro-therap*" or electrotherap*) W/2 ("Nucleus Accumbens" or "Subthalamic Nucleus" or brain or cortex or cortical or transcranial or cranial)) or tDCS or (deep W/ (rTMS or TMS))) TITLE (fatigue or mood* or sleep* or wakeful* or cognition or cognitive or apathy or fatigue or depressive or depression or mania or behavioral or neurobehavioural or neurobehavioral or psychiatric or neuropsychological or personality or appetite or weight gain or cardiovascular or gastrointestinal) TITLE-ABS-KEY (nonmotor or "non-motor") TITLE-ABS-KEY (parkinson*) INDEX(embase) OR INDEX (medline) OR PMID DOCTYPE(ed) OR DOCTYPE(bk) OR DOCTYPE(er) OR DOCTYPE(no) OR DOCTYPE(sh) OR DOCTYPE(ch) LANGUAGE (english) (TITLE-ABS-KEY ( ( alpaca OR alpacas OR amphibian OR amphibians OR animal OR animalOR antelope OR armadillo OR armadillos OR avian OR baboon OR baboons OR beagle OR beagles ORbee OR bees OR bird OR birds OR bison OR bovine OR buffalo OR buffaloes OR buffalos OR "c elegans" OR "Caenorhabditis elegans" OR camel OR camels OR canine OR canines OR carp OR cats OR cattle OR chick OR chicken OR chickens OR chicks OR chimp OR chimpanze OR chimpanzees OR chimps OR cow OR cows OR "D melanogaster" OR "dairy calf" OR "dairy calves" OR deer OR dog OR dogs OR donkey OR donkeys OR drosophila OR "Drosophila melanogaster" OR duck OR duckling OR ducklings OR ducks OR equid OR equids OR equine OR equines OR feline OR felines OR ferret OR ferrets OR finch OR finches OR fish OR flatworm OR flatworms OR fox OR foxes OR frog OR frogs OR "fruit flies" OR "fruit fly" OR "G mellonella" OR "Galleria mellonella" OR geese OR gerbil OR gerbils OR goat OR goats OR goose OR gorilla OR gorillas OR hamster OR hamsters OR hare OR hares OR heifer OR heifers OR horse OR horses OR insect OR insects OR jellyfish OR kangaroo OR kangaroos OR kitten OR kittens OR lagomorph OR lagomorphs OR lamb OR lambs OR llama OR llamas OR macaque OR macaques OR macaw OR macaws OR marmoset OR marmosets OR mice OR minipig OR minipigs OR mink OR minks OR monkey OR monkeys OR mouse OR mule OR mules OR nematode OR nematodes OR octopus OR octopuses OR orangutan OR "orang-utan" OR orangutans OR "orang-utans" OR oxen OR parrot OR parrots OR pig OR pigeon OR pigeons OR piglet OR piglets OR pigs OR porcine OR primate OR primates OR quail OR rabbit OR rabbits OR rat OR rats OR reptile OR reptiles OR rodent OR rodents OR ruminant OR ruminants OR salmon OR sheep OR shrimp OR slug OR slugs OR swine OR tamarin OR tamarins OR toad OR toads OR trout OR urchin OR urchins OR vole OR voles OR waxworm OR waxworms OR worm OR worms OR xenopus OR "zebra fish" OR zebrafish ) AND NOT ( human OR humans OR patient OR patients ) ) )

**Table 5:** Newcastle-Ottawa quality assessment scale for cohort studies † Kirsch-Darrow et al. [36] was a study combining cohort and RCT designs. Scoring explanation for each category *(a maximum of one star can awarded for each category with the exception of two stars in the comparability category)*: - Representativeness of the exposed cohort: A* - truly representative, B* - somewhat representative - Selection of the non-exposed cohort: A* - drawn from the same community as the exposed cohort, N/A - not applicable - Ascertainment of exposure: A* - secure records - Demonstration that outcome of interest was not present at start of study: A* - yes - Comparability: * study controls for one important factor, ** study controls for two important factors - Assessment of outcome: B* - record linkage - Follow-up: A* - adequate follow-up for outcomes to occur - Adequacy of follow-up of cohorts: B* - subjects lost to follow-up unlikely to introduce bias or description provided of those lost, C - no description provided, D - no statement

Cohort studies	Selection				Comparability	Outcome/Exposure			Quality score
	Representativeness of the exposed cohort	Selection of the non-exposed cohort	Ascertainment of exposure	Demonstration that outcome of interest was not present at start of study	Comparability of cohorts based on the design or analysis	Assessment of outcomes	Was follow-up long enough for outcomes to occur?	Adequacy of follow up of cohorts	
Ardouin et al. 1999 [30]	B*	N/A	A*	A*	**	B*	A*	D	7
Chen et al. 2019 [31]	A*	N/A	A*	A*	*	B*	A*	C	6
Dafsari et al. 2020 [32]	A*	N/A	A*	A*	*	B*	A*	B*	7
Hwynn et al. 2011 [33]	A*	N/A	A*	A*	**	B*	A*	D	7
Kirsch-Darrow et al. 2011 [36] †	A*	A*	A*	A*	*	B*	A*	D	7
Pillon et al. 2000 [35]	B*	N/A	A*	A*	**	B*	A*	D	7
Volkmann et al. 2009 [34]	B*	N/A	A*	A*	*	B*	A*	D	6

**Table 6:** Newcastle-Ottawa quality assessment scale for randomized controlled trials † Okun et al. [27] was labeled as an observational study. Scoring explanation for each category *(a maximum of one star can awarded for each category with the exception of two stars in the comparability category)*: - Is the case definition adequate? A* - yes, with independent validation, B - yes, with record linkage or based on self-reports - Representativeness of the cases: A* - consecutive or obvious representative series of cases - Selection of controls: A* - community controls - Definition of controls: N/A - not applicable - Comparability: * study controls for one important factor, ** study controls for two important factors - Ascertainment of exposure: A* - secure record - Same method of ascertainment for cases and controls - A* - Non-response rate: A* - same rate for both groups

Randomized controlled trials	Selection				Comparability	Exposure			Quality score
	Is the case definition adequate?	Representativeness of the cases	Selection of controls	Definition of controls	Comparability of cases and controls based on the design or analysis	Ascertainment of exposure	Same method of ascertainment for cases and controls	Non-response rate	
Celiker et al. 2019 [24]	A*	A*	A*	N/A	*	A*	A*	A*	7
Follett et al. 2010 [25]	A*	A*	A*	N/A	*	A*	A*	A*	7
Odekerken et al. 2013 [28]	A*	A*	A*	N/A	**	A*	A*	A*	8
Okun et al. 2009 [26]	A*	A*	A*	N/A	**	A*	A*	A*	8
Okun et al. 2014 [27] †	B	A*	A*	N/A	*	A*	A*	A*	6
Rothlind et al. 2007 [29]	A*	A*	A*	N/A	**	A*	A*	A*	8
Weaver et al. 2012 [13]	A*	A*	A*	N/A	**	A*	A*	A*	8
